# Performance evaluation of STANDARD Q COVID‐19 Ag home test for the diagnosis of COVID‐19 during early symptom onset

**DOI:** 10.1002/jcla.24410

**Published:** 2022-04-20

**Authors:** Hyoshim Shin, Seungjun Lee, Kristin Widyasari, Jongyoun Yi, Eunsin Bae, Sunjoo Kim

**Affiliations:** ^1^ Department of Laboratory Medicine Gyeongsang National University Hospital Jinju Korea; ^2^ 553954 Department of Laboratory Medicine Gyeongsang National University Changwon Hospital Changwon Korea; ^3^ Department of Laboratory Medicine Pusan National University School of Medicine Busan Korea; ^4^ Seegene Institute of Clinical Research Seegene Inc. Seoul Korea; ^5^ Gyeongsang National University College of Medicine Institute of Health Sciences Jinju Korea

**Keywords:** COVID‐19 testing, diagnosis, point of care, SARS‐CoV‐2

## Abstract

**Background:**

Surveillance and control of SARS‐CoV‐2 outbreak through gold standard detection, that is, real‐time polymerase chain reaction (RT‐PCR), become a great obstacle, especially in overwhelming outbreaks. In this study, we aimed to analyze the performance of rapid antigen home test (RAHT) as an alternative detection method compared with RT‐PCR.

**Methods:**

In total, 79 COVID‐19‐positive and 217 COVID‐19‐negative patients confirmed by RT‐PCR were enrolled in this study. A duration from symptom onset to COVID‐19 confirmation of <5 days was considered a recruiting criterion for COVID‐19‐positive cases. A nasal cavity specimen was collected for the RAHT, and a nasopharyngeal swab specimen was collected for RT‐PCR.

**Results:**

Sensitivity of the STANDARD Q COVID‐19 Ag Home Test (SD Biosensor, Korea), compared with RT‐PCR, was 94.94% (75/79) (95% [confidence interval] CI, 87.54%–98.60%), and specificity was 100%. Sensitivity was significantly higher in symptomatic patients (98.00%) than in asymptomatic (89.66%) patients (*p*‐value = 0.03). There was no difference in sensitivity according to the duration of symptom onset to confirmation (100% for 0–2 days and 96.97% for 3–5 days, respectively) (*p*‐value = 1.00). The RAHT detected all 51 COVID‐19 patients whose Ct values were ≤25 (100%), whereas sensitivity was 73.33% (11/15) among patients with Ct values >25 (*p*‐value = 0.01).

**Conclusion:**

The RAHT showed an excellent sensitivity for COVID‐19‐confirmed cases, especially for those with symptoms. There was a decrease in sensitivity when the Ct value is over 25, indicating that RAHT screening may be useful during the early phase of symptom onset, when the viral numbers are higher and it is more transmissible.

## 1 INTRODUCTION

Coronavirus disease 2019 (COVID‐19) was first reported in 2019 after a novel coronavirus, severe acute respiratory syndrome coronavirus 2 (SARS‐CoV‐2), was identified. Since its first discovery, SARS‐CoV‐2 has caused serious public health and economic concerns worldwide. The national strategy to combat COVID‐19 is based on rapid detection, isolation, contact tracing, and patient management to prevent the transmission of SARS‐CoV‐2.^1^ The detection of SARS‐CoV‐2 is the first step in assessing cases. It is also vital to detect SARS‐CoV‐2 as early as possible since SARS‐CoV‐2 may be coinfected with other microbial pathogens.^2^ Infection with SARS‐CoV‐2 may alter the human's microbiota, which further may affect the immune system.^3^ Based on a systematic review,^4^ a high prevalence of pathogenic microorganism was found among COVID‐19 patients. Thus, a delayed detection of SARS‐CoV‐2 could also result in increased mortality and morbidity due to the possibility of coinfection of SARS‐CoV‐2 and other pathogens.

A molecular diagnostic, that is, real‐time polymerase chain reaction (RT‐PCR) that has been widely used as a gold standard of detection depends on the sampling locations, probes of SARS‐CoV‐2 gene sequence that is used as a target for detection and days of symptom onset.^5^ Therefore, considering that RT‐PCR is complex, expensive, and slow to deliver,^6^ an alternative diagnostic method that is more user‐friendly and cost‐effective, which permits new cases to be isolated immediately, is in high demand.

The point‐of‐care (POC) diagnostic platform, which can provide results at the point of care instead of samples being sent to the laboratory, has been widely used and accepted as part of the control strategy for the restriction of COVID‐19.^7^ A lateral flow assay such as the antigen test is one of the most popular POC diagnostic platforms that have been widely studied and evidently plays some role in the restriction of COVID‐19 when a molecular diagnosis is difficult to perform.^7^, ^8^ Antigen tests (immunoassays) detect the presence of a specific viral antigen, mostly nucleocapsid protein, which strongly implies transmissible viral infection.^9^, ^10^ Antigen tests have a rapid turnaround time, whereby test results can usually be delivered within 5–30 min.^11^ Nevertheless, antigen tests for the diagnosis of SARS‐CoV‐2 are generally less sensitive than RT‐PCR.^12^ However, RT‐PCR, considered as the gold standard for SARS‐CoV‐2 diagnosis, can cause carryover contamination, requires expensive equipment and well‐trained technicians, and is costly to perform, which are challenges in resource‐poor countries.^13^, ^14^ In particular, molecular diagnosis takes several hours or a few days to obtain results and hampers the suspected cases’ immediate response.^15^ In such situations where COVID‐19 is overwhelming and medical personnel or diagnostic equipment is in short supply, a rapid diagnostic platform such as a rapid antigen home test (RAHT) could be considered a supplemental strategy.

Compared with conventional rapid antigen tests, a RAHT does not require personal precaution equipment, medical personnel, or visits to screening centers or hospitals. An RAHT is easy to use and cheaper than RT‐PCR. The RAHT may be used for school, business centers, or large gatherings to ensure safety from COVID‐19 transmission when the virus is widespread. The RAHT would also be helpful for, for example, those with disabilities, those in remote areas, or those in prisons, where medical services are difficult to access. In addition, those RAHT can be used at the point of care or at home. The Centers for Disease Control and Prevention (CDC) and World Health Organization (WHO) announced guidance for the optimal usage of antigen testing for SARS‐CoV‐2,^16^ but not for those RAHT. There has been no report on the performance of the RAHT in Korea yet. Therefore, we evaluated the diagnostic performance of the RAHT with a consideration of clinical characteristics, including the presence of symptoms, days after the symptom onset, and the Ct value of the RT‐PCR.

## 2 MATERIALS AND METHODS

### 2.1 Subjects

This study was conducted among 79 SARS‐CoV‐2‐infected patients and 217 non‐infected patients confirmed by RT‐PCR. Patients infected with SARS‐CoV‐2 were admitted to COVID‐19‐designated hospitals or institutes in June and July 2021. These patients were either mildly symptomatic or asymptomatic. Non‐infected patients were outpatients or were admitted for other medical conditions at Gyeongsang National University Changwon Hospital (GNUCH). All participants submitted written informed consent. This study was approved by the institutional review board (IRB) of GNUCH (IRB No. 2021–04–019).

### 2.2 Specimen collection

The specimens for detection of SARS‐CoV‐2 were self‐collected by each participant after blowing the nose and inserting a flocked swab into nostril at a depth of 1 to 2 cm and rotating three times against the surface of the nasal cavity, according to the manufacturer's manual.

### 2.3 Inclusion and exclusion criteria

In this study, the RT‐PCR‐positive tested group included patients with mild symptoms or without symptoms. Among them, about a third (1/3) were asymptomatic. The negative group included patients who visited GNUCH but the RT‐PCR test result came back negative. In addition, patients who took antiviral drugs for COVID‐19 were excluded. Healthcare workers or experts who had experience for in vitro diagnostic equipment, such as glucometer, were also excluded from the study.

### 2.4 Rapid antigen home test

The STANDARD Q COVID‐19 Ag Home Test (SD Biosensor, Suwon, Korea) is an RAHT that qualitatively detects the presence of the SARS‐CoV‐2 nucleocapsid protein in human nasal specimens via chromatographic immunoassay. The STANDARD Q COVID‐19 Ag Home Test, hereinafter referred to as the SD Q home test, is a detection kit that allows the entire procedure to be conducted at home. The SD Q home test cassette is coated with two lines, that is, a control line (C) and a test line (T). When the specimen contains SARS‐CoV‐2, the antigens will bind to the SARS‐CoV‐2‐specific antibodies coated on the test line region (T), which later will generate a colored line on the test strip. If the specimen does not contain SARS‐CoV‐2 antigens, a colored line will not appear in the T region. The result was interpreted as positive if two lines appeared on the nitrocellulose membrane.

### 2.5 Real‐time reverse‐transcription polymerase chain reaction (RT‐PCR)

To compare the sensitivity analysis of the SD Q home test, RT‐PCR assay for the qualitative detection of SARS‐CoV‐2 nucleic acids was used to detect the presence of SARS‐CoV‐2 RNA‐dependent RNA polymerase gene (*RdRp*) in the samples. Specimens for RT‐PCR were collected from the nasopharyngeal and were collected at the same time as the nasal swab specimens used for the SD Q home test. The result was interpreted as positive only if the cycle threshold (Ct) value of *RdRp* was within the cutoff, according to the manufacturer's recommendation.

### 2.6 Statistical analysis

Fisher's exact test^17^ was used to evaluate the differences in SD Q home test sensitivity according to the presence of symptoms, the duration between symptom onset and the confirmation date for asymptomatic patients, and the Ct values of RT‐PCR, which indicate viral load. The correlation of the days after symptom onset and the Ct values of *RdRp* was evaluated using Spearman's correlation analysis.^18^, ^19^ A *p*‐value of <0.05 was considered statistically significant. We performed all statistical analyses using the SAS software ver. 9.4 (SAS Institute Inc., Cary, NC) and R version 3.6.3 (R Foundation for Statistical Computing, Vienna, Austria).

## 3 RESULTS

### 3.1 Sensitivity of SD Q home test compared with RT‐PCR

The overall sensitivity of the SD Q home test was 94.94% (75/79) (95% CI, 87.54%‐98.60%), with total specificity was 100% compared with the RT‐PCR (217/217), the positive predictive value (PPV) was 100% and the negative predictive value (NPV) was 98.19% (Table 1). Furthermore, we found that the sensitivity of the SD Q home test in the symptomatic patients (98.00%) was significantly higher than in the asymptomatic patients (89.66%) (*p*‐value = 0.03) (Table 2).

**TABLE 1:** Diagnostic performance of the rapid antigen home test

Diagnostic performance	Value	95% CI
Sensitivity, %	94.94%	87.54%–98.60%
Specificity, %	100.00%	98.31%–100.00%
Positive predicted value, %	100.00%	100.00%–100.00%
Negative predicted value, %	98.19%	95.43%–99.30%
Accuracy, %	98.65%	96.58%–99.63%

Abbreviation: CI, confidence interval.

**TABLE 2:** Sensitivity of rapid antigen home test according to the presence of symptoms, days after the symptom onset, and the Ct value of the RT‐PCR

Clinical Characteristics	RAHT (+)	RAHT (−)	Sensitivity % (95% CI)	*p*‐value
Overall	75	4	94.94% (87.54%–98.60%)	0.03
With symptoms	49	1	98.00% (89.35%–99.95%)	
Without symptoms	26	3	89.66% (72.65%–97.81%)	
Days after symptom onset				
No. of analyzed	49	1	98.00% (89.35%–99.95%)	1.00
0–2	17	0	100% (80.49%–100.00%)	
3–5	32	1	96.97% (84.24%–99.92%)	
Ct values^a^				
No. of analyzed	75	4	94.94% (87.54%–98.60%)	0.01
Ct≤20	51	0	100% (93.02%–100.00%)	
20<Ct≤25	13	0	100% (100.00%–100.00%)	
25<Ct	11	4	73.33% (44.90%–92.21%)	

Abbreviations: CI, confidence interval; Ct, cycle threshold; RAHT, rapid antigen home test; RT‐PCR, real‐time polymerase chain reaction.

^a^ Ct value for the *RdRp* gene in a RT‐PCR assay.

### 3.2 Sensitivity of SD Q home test according to the days after symptom onset (DSO)

Evaluation on the sensitivity of SD Q home test according to the DSO demonstrated that there is no significant difference in sensitivity between a test conducted at 0–2 days (17/17, 100%) and one conducted at 3–5 days (32/33, 96.97%) after symptom onset (*p*‐value = 1.00), suggesting that 0–5 DSO is the optimal time to perform an RAHT.

### 3.3 Sensitivity of SD Q home test according to the Ct value

The sensitivity of SD Q home test was evaluated by restricting Ct values of RT‐PCR‐positive diagnosed specimens into three groups, that is ≤20, 20 < Ct ≤25, and 25 < Ct. The sensitivity of the SD Q home test was up to 100% for the specimens obtained from patients where Ct ≤20 (51/51) or 20 < Ct≤25 (13/13). For patients where 25<Ct, the sensitivity of the RAHT declined to 73.33% (11/15) (*p*‐value = 0.01) (Table 2).

### 3.4 Correlation between Ct values of symptomatic patients and the DSO

**FIGURE 1:**
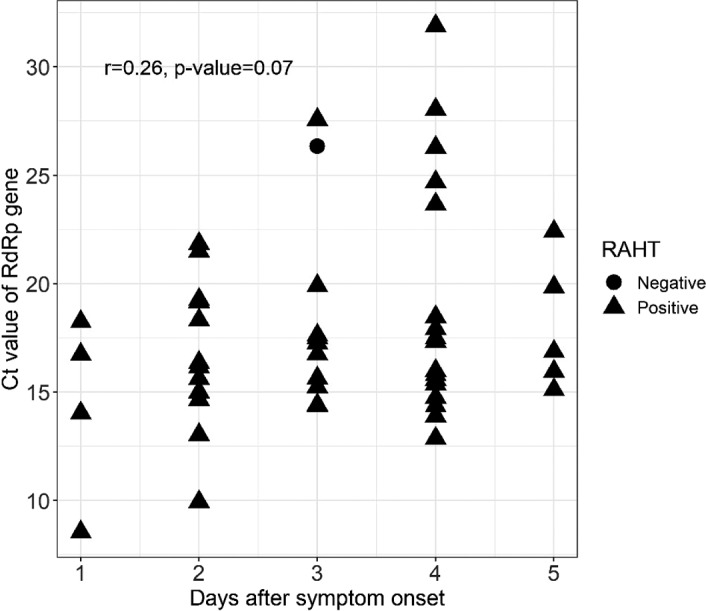
Relationship between the days after symptom onset and the cycle threshold (Ct) values of SARS‐CoV‐2 RNA‐dependent RNA polymerase gene (*RdRp*) from symptomatic patients (*N* = 50)

In this study, we observed that the Ct values increased as the DSO increased (Figure 1). Nevertheless, correlation analysis between Ct values and DSO by Spearman's correlation test showed a 'marginally significant' correlation (*p*‐value = 0.07).

## 4 DISCUSSION

In the present study, we evaluated the sensitivity of the SD Q home test according to the presence of symptoms, days after the symptom onset or the confirmation date for asymptomatic patients, and the Ct values of the RT‐PCR. Accordingly, our results showed consistently with the current FDA guidelines, which mention that a rapid COVID‐19 home test should have a specificity of at least 98% but can have a sensitivity as low as 80%.^20^

Our finding also demonstrated higher sensitivity of the SD Q home test in the symptomatic patients (98.00%) compared with asymptomatic one (89.66%). A similar study conducted in the USA also discovered the higher sensitivity of the rapid antigen test for symptomatic populations compared with asymptomatic population.^21^ This finding is reasonable because symptomatic patients are likely to have a high accumulation of virus load in the body, leading to positive results upon detection. Nevertheless, considering that, even in an asymptomatic patient, the sensitivity value of the RAHT that we used in our study is up to 89.66%, we are optimistic that the SD Q home test is a handy initial screening test to monitor the spread of COVID‐19 in the community.

Ct values are considered an indicator of viral load, represented by the nucleic acid in the sample.^22^, ^23^ Higher Ct values indicate a lower amount of nucleic acid (viral load), while lower Ct values indicate a higher amount of nucleic acid.^22^, ^23^ Accordingly, we evaluated whether the sensitivity of the SD Q home test is influenced by viral load. We confirmed that sensitivity of SD Q homes test was at its highest when the Ct values ≤25. In our study, there are four specimens that have Ct value of 30<Ct. Among four of them, only one showed positive result when we tested using RAHT. Our result corresponds with previous studies reporting that the sensitivity of the RAHT is inversely correlated with Ct values.^24^, ^25^

We also evaluated the sensitivity of the SD Q home test according to DSO and demonstrated that 0–5 DSO is the optimal time to perform an RAHT. Consistently, a previous study also reported that the sensitivity of the rapid antigen test was higher within 7 DSO compared with a population with extended days of symptoms.^21^ Given that the sensitivity of the SD Q home test is at the highest level in symptomatic patients within 5 DSO with a lower Ct value, we concluded that viral load can be categorized as the most important factor for determining RAHT sensitivity.

Analyzes of the correlation between Ct values and DSO by Spearman's correlation test showed a 'marginally significant' correlation (*p*‐value = 0.07). We assumed this is due to the narrow range of DSO (0–5 days). At early infection, viral accumulation tends to be high while the Ct values are lower.^22^, ^26^ Hence, at 0–5 DSO, no significant changes in Ct values were observed due to the constantly high level of viral load, while the sensitivity of the RAHT is at the highest level during these periods of time. Extending the range of DSO may allow for a more accurate evaluation of the correlation between Ct value and DSO. However, taking all these results into consideration, we expect that the RAHT may be practical as a diagnostic tool during the early phase of symptom onset when viral load is in great number and it is more transmissible.

However, we are also aware of the presence of several studies reporting the poor performance of rapid antigen tests, including the RAHT.^27^, ^28^ The difference in the performance and sensitivity of rapid antigen tests, including the RAHT, may be due to such factors as the type of samples, the type of assays, the time of sample collection, the accuracy of sampling, and the transport and storage.^21^, ^29^

Finally, the RAHT is increasingly used for screening COVID‐19 because it is low‐cost and available at points of care and does not require well‐trained technicians to administer. Many studies have been initiated to evaluate the effectiveness of an antigen test application compared with RT‐PCR.^30^, ^31^, ^32^ Most of these studies are in concordance with our study, which demonstrated the effectiveness and benefits of a rapid antigen test, that is, the RAHT, in offering robust detection of COVID‐19 that complements molecular detection.

The narrow range of days after symptom onset may become a limitation in our study; nonetheless, we demonstrated that the SD Q home test revealed a reasonably good performance compared with RT‐PCR, especially in symptomatic patients. This finding suggests that the SD Q home test is quite helpful as an alternative diagnostic tool in situations where a significant number of people in a specific group are suspected of having SARS‐CoV‐2 and molecular diagnosis technology or expertise are limited. As there was also a decrease in sensitivity with increments in Ct values and days after symptom onset, we conclude that the SD Q home test might be more beneficial for screening at the early phase of infection, when the viral numbers are higher or it is in a more infectious stage.

## CONFLICT OF INTEREST

No potential conflict of interest was reported by the authors.

## AUTHOR CONTRIBUTIONS

J.Y, E. B, and S. K involved in conceptualization. S. L involved in sample collection and experiment. H. S involved in writing–original draft preparation. K.W and S. K involved in writing–review and editing. S. K. involved in supervision and funding acquisition. All the authors have read and agreed to the final version of the article.

## Data Availability

Data of this study can be available on reasonable request.
